# Lactic acid fermentation of food waste at acidic conditions in a semicontinuous system: effect of HRT and OLR changes

**DOI:** 10.1007/s13399-022-03201-w

**Published:** 2022-08-20

**Authors:** Simone Pau, Lea Chua Tan, Sonia Arriaga, Piet N. L. Lens

**Affiliations:** 1 National University of Ireland, University Road, GalwayGalway, Ireland; 2 Environmental Sciences Department, Instituto Potosino de Investigación Científica Y Tecnológica, San Luis Potosí, Mexico

**Keywords:** Reactor fermentation, Lactic acid, Food waste, Valorization, Acidic environment

## Abstract

Lactic acid production through fermentation is an established technology, however, improvements are necessary to reduce the process costs and to decrease its market price. Lactic acid is used in many industrial sectors and its market has increased in the last decade for its use as the raw material for polylactic acid product. Using food waste as a cheap and renewable substrate, as well as fermentation at uncontrolled pH, helps to make the production cheaper and to simplify the downstream purification process. Lactic acid production at acidic conditions and the role of varying organic loading rate (OLR) and hydraulic retention time (HRT) were tested in two different semicontinuous batch fermentation systems. Reactor performances indicated that lactic acid fermentation was still possible at pH < 3.5 and even up to a pH of 2.95. The highest lactic acid production was recorded at 14-day HRT, 2.14 g VS/L·day OLR, and pH 3.11 with a maximum lactic acid concentration of 8.72 g/L and a relative yield of 0.82 g lactate/g carbohydrates. The fermentation microbial community was dominated by *Lactobacillus* strains, the organism mainly responsible for lactic acid conversion from carbohydrates. This study shows that low pH fermentation is a key parameter to improve lactic acid production from food waste in a semicontinuous system. Acidic pH favored both the selection of *Lactobacillus* strains and inhibited VFA producers from utilizing lactic acid as primary substrate, thus promoting the accumulation of lactic acid. Finally, production yields tend to decrease with high OLR and low HRT, while lactic acid production rates showed the opposite trend.

## Introduction

Over 30% of the food produced in the world becomes a waste with food waste (FW) and food loss (FL) generated along every step of the food supply chain [1]. According to the Food and Agriculture Organization of the United Nations, 1.3 billion tons of food are converted to waste every year [2]. One of the alarming environmental impacts associated with FW and FL generation is the production of greenhouse gases (GHG) with emissions increasing at 2.4% per year [3] since 1961 reaching nowadays 3.3 gigatons of CO_2_ per year. This corresponds to 6% of the total global anthropogenic GHG produced in one year [4]. To avoid the increasing problems connected to FW and GHG production, prevention of its generation is the most desirable route, but when FW cannot be prevented, a correct treatment and management strategy is needed. The classical management methods of FW have always been through landfill disposal, incineration and/or composting [5]. The first two do not solve the environmental aspects due to the impacts associated to landfilling (biogas generation, risk of groundwater pollution) and thermo-valorization (incomplete oxidation of the organic matter with formation of harmful compounds for human health), while the third approach produces a material that is not economically competitive [5].

An alternative solution to classical methods is the use of biological conversion processes to valorize FW and generate value-added products. Due to its organic material richness and high biodegradability [6], FW serves as a perfect platform to produce chemical commodities and energy through its biological conversion [7]. This can be achieved through anaerobic digestion (AD) or dark fermentation (DF) processes, both of which have been shown effective for waste stream valorization in the production of several commodities like methane [6, 8], hydrogen [9, 10], organic acids (volatile fatty acids (VFAs) [11], lactic acid (LA) [12]), and solvents (ethanol) [13]. Among these chemicals, LA is getting more and more attention of researchers and industries.

LA is one of the intermediates that can be formed during acidogenesis in an AD process. LA is a water miscible acid that presents two different isomers D-LA and L-LA used in several applications such as foods, pharmaceuticals, cosmetics, and chemical industries [14]. Among the many applications, LA is gaining market attention in the last decade for being the main feedstock to produce the most used bioplastic in the world, namely polylactic acid (PLA) [15]. PLAs are produced chemically through a LA polymerization process and can potentially replace fossil-based plastics [15]. However, for PLA to be competitive against petroleum-based polymers, the LA price needs to be reduced by 50% [14].

Although LA can be produced chemically, almost 90% of industrial LA production is obtained through biological fermentation of carbohydrates [16]. LA is commonly produced in batch fermentation under mesophilic conditions and neutral pH using pure starting substrates and inoculum such as glucose/xylose and *Lactobacillus* cultures, respectively [14, 17]. In addition to this, to maintain a neutral pH during fermentation, alkalinizing agents such as calcium oxide/sodium oxide are added to the broth [18]. This permits the conversion and precipitation of LA to lactate salts in the form of calcium or sodium lactate which can be extracted from the system in solid form. To complete the process, lactate salts are commonly acidified back with sulfuric acid to get pure LA which subsequently produces gypsum as waste material that must be managed and disposed [18, 19]. Overall, the current LA value stands at 3.00–4.00 USD/kg [16] which is estimated to increase 19% per year and predicted to have a global market that will reach 9.8 billion USD by 2025 [20]. Despite the established process for the industrial production of LA, the necessity to reduce its price mandates for research to improve the process and find alternatives in order to cut the operational costs.

Process areas that can be targeted for improvement are through utilizing a more sustainable substrate and maximizing reactor parameters. For the substrate, waste streams such as FW represent a cheap and renewable alternative [21, 22] to the pure substrates commonly utilized for LA production, which accounts for 40–70% of the total production cost [18]. For reactor parameters, another route that has yet to be fully investigated is operating at acidic environments of below the pKa of LA 3.78 [18]. It has been established that pH is one of the key parameters in fermentation processes. During acidogenesis, the pH tends to decrease naturally and common practice at industrial scale is to maintain a high pH during operation. This is done through constant dose of alkalinity (calcium hydroxide and sodium hydroxide) which accounts for almost 15% of the total cost of the process [23]. This addition turns LA into a lactate salt present in the solid phase. The salt can be easily separated from the fermentation broth but requires long and expensive downstream processes to purify the LA. Working under uncontrolled pH conditions, without using neutralizing agents, is still a challenge [18]. The advantages of working at low pH include the following: (i) according to Itoh et al., pH 3.5 is optimal for LA bacteria selection, inhibiting VFAs producers that could compete for the primary substrate or use LA for acid conversion [24] and (ii) operating at low pH negates the need to add neutralizing agents providing an opportunity to decrease costs related to the reagents and to simplify the extraction and purification phase since LA can be extracted directly in the liquid form and better extraction methods like electrodialysis, solvent, adsorption, and ion exchange resins can be applied [18, 23, 25].

Despite the clear advantages brought by the low pH fermentation listed above, there is currently limited information on the performance of continuous and semicontinuous systems for LA fermentation using FW as the substrate at these acidic conditions. This study is a continuation from the previous work [26] and aims to investigate LA fermentation under uncontrolled acidic systems in two different 1L upflow anerobic sludge bed (UASB) reactors operated in semicontinuous batch mode configuration. The role and effect of the hydraulic retention time (HRT) and organic loading rate (OLR) variations on FW fermentation were also explored. The performance of the process was evaluated in terms of LA conversion yields, LA production rates, by-product formation, and with the microbial community changes during the different phases of the experimental run.

## Methodologies

### Substrate

Synthetic FW was used as the substrate for both the screening batch tests and the semicontinuous reactor run. The FW recipe was reported previously [26] and taken from the study of Ariunbataar et al. [27] with the aim to mimic the average household FW in Europe. The FW was prepared by reducing ingredients into small pieces, cooked, mixed in the right proportion, and finally milled with the addition of 200 ml of water in order to obtain a homogenous slurry. The FW stock was stored at − 21 °C when not in use and at 4 °C after defrosting when feeding into the reactor. FW composition (combined solid and liquid organic fractions) were fully characterized with the results reported in Table 1.

**Table 1:** Characterization of the food waste used in this study

Property	Parameter	Value
Solid content	TS [% wet based]	17.08 ± 0.08
	VS [% wet based]	16.41 ± 0.08
	VS [% TS]	96.08 ± 0.01
Composition	Total COD [g/kg]	152.9 ± 6.09
	Total Carbohydrates [g/kg]	64.98 ± 2.65
	Total Protein [g/kg]	11.43
	Ammonia [mg/L]	7.90 ± 2.00
Physical properties	pH	4.72
	Conductivity [mS/cm]	4.21
	Salinity [g/kg]	2.10

### Inoculum screening test

Prior to the semicontinuous reactor experimental run, an initial batch screening test was conducted. Three different microbial community sources were tested: two external inocula and the indigenous bacterial community present in the FW (as control). The first inoculum was an aerobic primary sludge (PS) (total solids (TS) = 3.58%, volatile solids (VS) = 2.44%) taken from the oxidation tank used to treat municipal wastewater (Tuam, Galway Co., Ireland). The second inoculum was a commercial yogurt (TS = 13.71%, VS = 12.81%) bought in the supermarket (Dunnes stores, Dublin, Ireland).

Three identical 1L UASB reactors (0.7 L working volume) were set up for an initial batch screening test. The reactors were operated in batch mode with incubation carried out at 37 °C for 9 days. Agitation was provided through an internal recirculation line (upflow flow rate of 230 ml/min) that allowed liquid mixing. 1 L gas bags were attached on the top port of the reactor to collect biogas produced during the fermentation. pH was constantly monitored with a pH meter but uncontrolled and allowed to drop during acidogenesis.

The UASB reactors were operated with different microbial community sources and FW: primary sludge (B1) and yogurt (B2) and FW alone as control (no external addition inoculum provided – B3). Both B1 and B2 were inoculated 24 h prior to the FW feeding at 2 g VS/L of sludge and yogurt, respectively, for bacterial acclimation. All three reactors were then fed with 8 g VS/L of food waste and flushed with nitrogen gas for 15 min to maintain anaerobic conditions. Samples were taken daily and analyzed for chemical characterization (chemical oxygen demand (COD), total carbohydrates, volatile fatty acids (VFAs) and LA).

### Semicontinuous fermentation

**Fig. 1:**
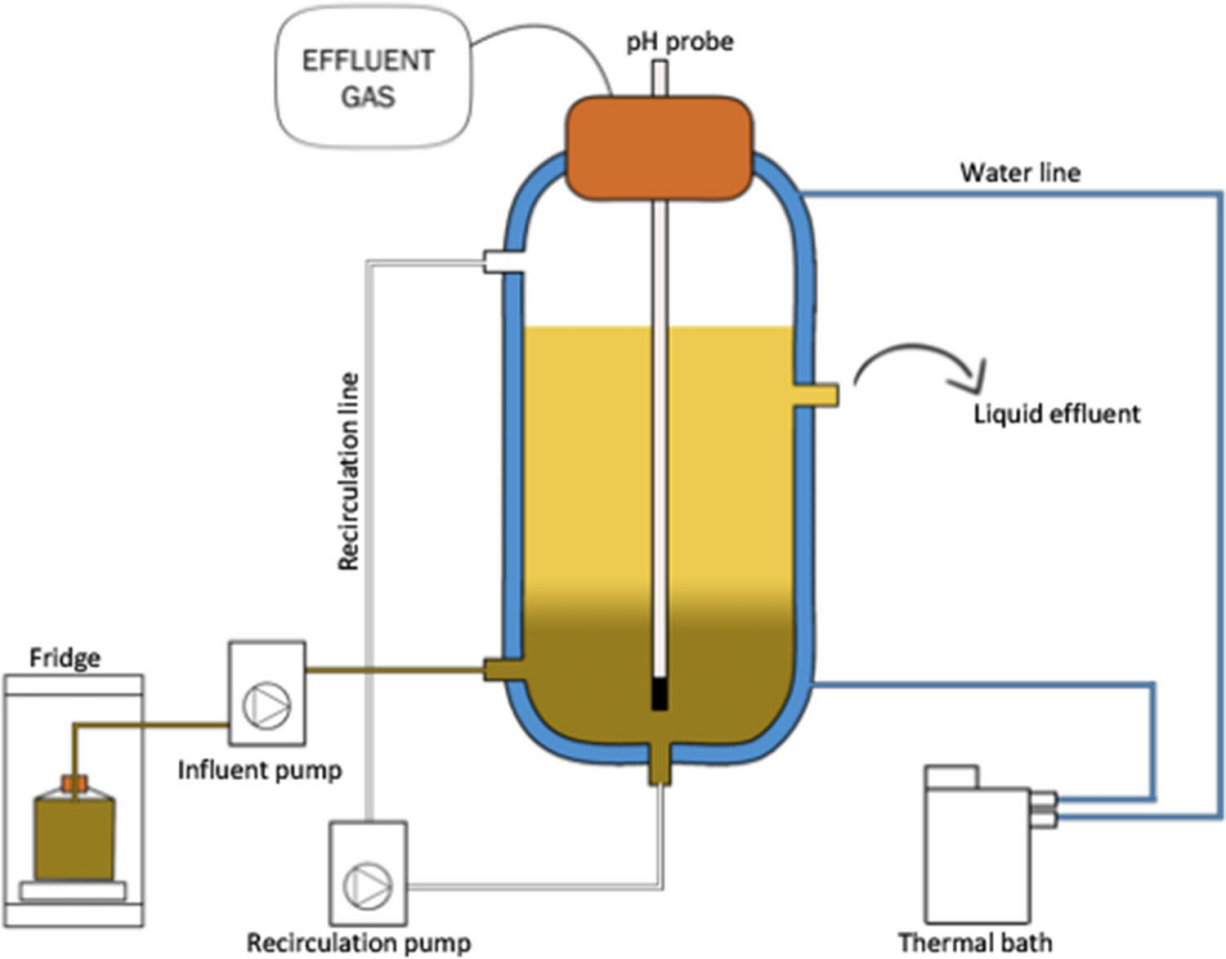
Schematic diagram of semicontinuous the reactor used in this study

The 1L UASB reactors used for the initial batch screening test were modified to operate under semicontinuous mode with intermittent FW feeding (Fig. 1). The reactors R1 and R2 were setup and operated at the same HRT, but at different OLR (Table 2). The fermentation in each reactor was carried out at mesophilic conditions (37 °C) for 128 days following a repeated batch configuration. The reactors were started with different initial FW concentrations of 8 g VS/L (R1) and 15 g VS/L (R2) and inoculated with 2 g VS/L of yogurt (best result based on the initial batch screening test). Every batch cycle lasted half the duration of the HRT with an exchange ratio of 0.5. At the end of every cycle, 350 mL of fermentation broth was discharged, and 350 mL of new substrate was fed to restore the initial solid concentration.

**Table 2:** Operating conditions of the semicontinuous reactors (R1 and R2) during the different fermentation phases

Phase	Time [day]	Duration [day]	N° of batch cycles	HRT* [day]	OLR** [g VS/L·day]
I	0–28	28	6	18 → 4	-
II	28–44	16	7	4	R1: 4.00
					R2: 7.50
III	44–92	48	7	14	R2: 1.14
					R2: 2.14
IV	92–128	36	9	8	R1: 2.00
					R2: 3.75

^*^HRT, hydraulic retention time

^**^OLR, organic loading rate

The gas contained in the headspace was internally recirculated to guarantee constant mixing and solid suspension. pH was left uncontrolled but monitored throughout the experiment. One-liter gas bags were attached to the top port of the reactor to collect the biogas produced.

The role of HRT and OLR changes was evaluated in this experiment. Three different HRT (4, 8, 14 days) and six different OLR (1.14, 2.00, 4.00 g VS/L day for R1 and 2.14, 3.75, 7.5 g VS/L for R2) were tested. The experimental run was divided in 4 main phases based on the HRT of the reactors as shown in Table 2. The reactors were operated in batch mode for the first 9 days. From days 10 to 28, the HRT was gradually reduced from 18 to 4 days in order to progressively increase the organic loads in ingress. From days 29 to 44 (phase II), the HRT was kept constant at 4 days. From days 44 until 92 (phase III), the HRT was increased to 14 days due to the low LA conversion yields detected in phase II, especially in R2. Finally, an HRT of 8 days was set from day 92 until the end of the operational run. Yogurt inoculation was made at the beginning of every phase.

Liquid samples (2 mL) were taken daily for chemical analysis (LA, VFA, carbohydrates, and chemical oxygen demand (COD)). At the end of every cycle, samples were taken for TS and VS content determination. At the end of every phase (days 0, 28, 44, 92, 128), samples were taken for the microbial community analysis.

### Analytical analysis

Samples were centrifuged and filtered using either 0.45 µm or 0.22 µm cellulose acetate filters (depending on analysis conducted). Filtered samples were analyzed for soluble chemical oxygen demand (sCOD), carbohydrates, LA, and VFA. sCOD analysis was performed using an AA3 continuous flow nutrient analyzer (Seal Analytical, King’s Lynn, United Kingdom). Carbohydrates, LA, and VFAs (C_2_ to C_5_) were measured using a 1260 Infinity II Agilent Technologies high-performance liquid chromatograph (HPLC) (Agilent, Santa Clara, USA) with a Hi-Plex H 300 mm × 7.7 mm column and detected with a refractive index detector (2.31 Hz) at 55 °C. The mobile phase was a 0.5 mM solution of H_2_SO_4_ at a flow rate of 0.7 mL/min with an injection volume of 50 µl [25].

The TS content was determined by drying the samples at 105 °C overnight and the VS content to 550 °C for 2 h following the procedure described by *APHA 2012* [28]. pH was determined with a pH-meter 300 pH/ORP (Cole Parmer, Vernor Hill, USA) and an electron probe VWR Thin, ceramic junction (VWR, Radnor, USA).

### Microbial community analysis

Triplicate samples were taken from both reactors at the end of every phase (days 0, 28, 44, 92, 128) to characterize the bacterial microbial community. DNA was extracted for each phase in triplicate using Qiagen DnNeasy Power Soil extraction kits (QIAGEN, Hilden, Germany) according to the manufacturer's protocol. Purity and concentration of the extracted DNA were analyzed using a NanoDrop XXX (ThermoFisher, Walthan, MA) and QuBit fluorometer (Invtrogen, Carlsbad, CA, US). Extracted DNA samples were kept at − 20 °C prior to sequencing. Extracted DNA samples were sent to Novogene (Cambridge, UK) for sequencing using the NovaSeq 6000 platform. Bacterial DNA sequences were performed using the universal bacterial primers 515F and 806R. Sequencing raw data was processed by Novogene and the microbial community results were provided as % relative abundance.

### Calculations and statistical analysis

Reactor performance analysis was carried out for every batch cycle (i) per phase over the course of the semicontinuous run to calculate for the LA production, carbohydrate consumption, and organic composition. All concentrations were expressed in terms of g COD/L. For every cycle, the following functions were calculated:

1 $${LA}_{p}{(t)}_{(i)}= {LA(t)}_{\left(i\right)}- {LA({t}_{0})}_{\left(i\right)}$$

where $\left(i\right)$ is the number of the cycle; ${LA}_{p}(t)$ is the LA produced at time (t); ${LA(t)}_{\left(i\right)}$ is the LA concentration at time (t); and ${LA({t}_{0})}_{\left(i\right)}$ is the LA concentration in the beginning of the cycle (t_0_).

2 $${CA}_{c}{(t)}_{(i)}= {CA({t}_{0})}_{\left(i\right)}- {CA(t)}_{\left(i\right)}$$

where ${CA}_{c}{(t)}_{(i)}$ are the carbohydrates consumed at time (t); ${CA({t}_{0})}_{\left(i\right)}$ is the carbohydrate concentration at the beginning of the cycle (t_0_); and ${CA(t)}_{\left(i\right)}$ is the carbohydrate concentration at time (t).

3 $${LA}_{y}{(t)}_{(i)}= \frac{{LA}_{p}{(t)}_{(i)}}{{CA}_{c}{(t)}_{(i)}}$$

where ${LA}_{y}{(t)}_{(i)}$ is the LA yield calculated as the ratio of [1] and [2] (g LA_(COD)_/g CA_(COD)_).

The calculations per cycle were then grouped together based on the phase (*p*) and averaged in order to evaluate the influence of the HRT and OLR in the process efficiency. The averages were calculated as follows:

4 $${LA}_{p}{(t)}_{(p)}= \frac{\sum_{i=0}^{Nc(p)}{LA}_{p}{(t)}_{(i)}}{{Nc}_{(p)}}$$

5 $${CA}_{c}{(t)}_{(p)}= \frac{\sum_{i=0}^{Nc(p)}{CA}_{C}{(t)}_{(i)}}{{Nc}_{(p)}}$$

6 $${LA}_{y}{(t)}_{(p)}= \frac{\sum_{i=0}^{Nc(p)}{LA}_{y}{(t)}_{(i)}}{{Nc}_{(p)}}$$

where (*p*) is the number of the phase (*p* = 2, 3, 4) and Nc_(p)_ is the number of the cycles in phase p.

The average yield/points per phase were plotted and used as the base to build the theorical curves to evaluate the influence of HRT and OLR for LA production using a kinetic model. LA production curves were fitted using a 3 parameter Gompertz model following the form indicated by Germec et al.[29]:

7 $${LA}_{y}{(t)}_{(p)} = {LA}_{Th (p)}\times \mathrm{exp }(-\mathrm{exp }(-\mathrm{kLA}(\mathrm{p}) \times (\mathrm{t}-\mathrm{tc}(\mathrm{p}))$$

where ${LA}_{y}{(t)}_{(p)}$ is the average LA production yield at time (t); ${LA}_{Th (p)}$ is the theoretical LA yield; k_LA(p)_ is the kinetic constant; and t_c(p)_ represents the lag phase. The averaged data were fitted using the Origin 2018 software (v. 9.5.95, Norhampton, USA) and the resulting coefficient of determination R^2^ was used to determine the quality of data fitting for each experimental dataset.

## Results

### Influence of inoculum on LA fermentation (batch screening test)

**Fig. 2:**
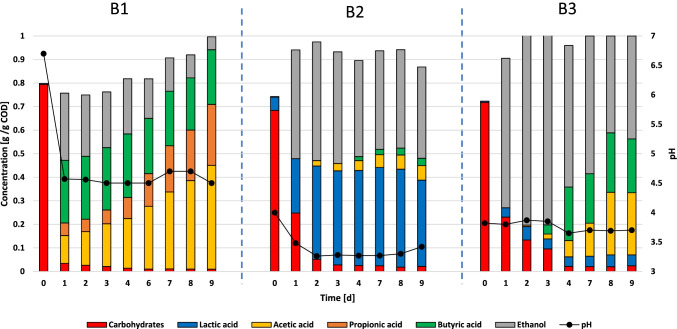
Organic composition in terms of sCOD percentage and pH profile obtained through batch FW fermentation with primary sludge (B1), yogurt (B2), and the indigenous FW microbial community (B3)

Figure 2 shows the results of the three screening batch incubations. The concentration is expressed in terms of grams of compound (converted in COD) per gram of sCOD. This represents the percentage composition of the fermentation broth during the experimental run. The pH variation is shown as well.

The primary sludge incubation (B1) started with a pH of 6.7 indicating the alkalizing effect of the sludge on the FW. On the other hand, the yogurt bacteria incubation (B2) and the control (B3) started from a lower pH of 4.00 and 3.82, respectively. A rapid decrease in pH was observed for reactor B1, with the pH dropping 2.13 units in 24 h at 4.57 and remained relatively stable until the end of the batch run. Reactor B2 showed a gradual but constant pH decrease from 4.00 to 3.26 within day 2 followed by a slightly increase and a final value of 3.42 on day 9. On the other hand, the pH in reactor B3 was constant for the first 3 days at approximately 3.80 with only a slight decrease to 3.70 by the end of the run.

The carbohydrate degradation occurred quickly on the first day of incubation in reactor B1 with 95.6% of the initial substrate converted to VFAs. Slower consumption yields were observed in B2 and B3. Reactor B2 presented a 63.6% carbohydrates degradation after day 1 before reaching 95.9% at day 3. Similarly, reactor B3 had 67.8% degradation in the first day and reached 96.9% at day 4.

FW fermentation behaved different among the three batch reactor incubations. As shown in Fig. 2, the different microbial ecology inoculated in each reactor resulted in a diverse metabolite composition produced. The common metabolic products were LA, ethanol, acetate, propionate, and butyrate. LA production was consistent in reactor B2, with 0.41 g/g COD converted at day 7 and a final concentration of 0.37 g/g COD (Fig. 2–B2). Opposite to this, reactor B3 only produced 0.05 g/gCOD LA while none LA was detected in B1.

Table 3 reports the composition of the fermentation broth at the end of the experimental run. B1 presented a very heterogenous metabolic composition. VFAs and ethanol were produced within the first day with a concentration of 0.44 and 0.28 g/g COD, respectively. From day 2 until the end of the experiment, VFAs concentrations increased constantly reaching 0.93 g/g COD, while ethanol instead decreased to a final value of 0.05 g/g COD (Fig. 2 – B1). In incubation B2, ethanol was produced and maintained throughout the operation, reaching a maximum concentration of 0.50 g/g COD by day 2. On the other hand, VFA production was nearly inhibited with only traces (3%) of acetate and butyrate (Fig. 1 – B2). B3 showed a steady production of ethanol in the first three days reaching a concentration of 0.83 g/g COD by day 3. This was followed by a simultaneous ethanol degradation and VFA (acetate and butyrate) production registering a final concentration of 0.44 and 0.49 g/g COD, respectively, by the end of the experimental run (Fig. 2 – B3).

**Table 3:** Organic composition and final pH for the batch screening tests (B1–B3) at the end of the experimental run (day 9). The percentages (%) are expressed as the COD fraction of the product over the total sCOD

	B1	B2	B3
Final pH	4.50	3.73	3.70
Residual carbohydrates [%]	0.9	2.1	2.4
Lactic acid [%]	-	36.6	4.6
Ethanol [%]	5.5	38.8	43.7
Acetic acid [%]	44.0	6.2	26.4
Propionic acid [%]	25.9	-	-
Butyric acid [%]	23.2	3.1	22.9
Total VFAs* [%]	93.1	9.3	49.3

^*^VFAs, volatile fatty acids

### Semicontinuous fermentation

#### pH profile

**Fig. 3:**
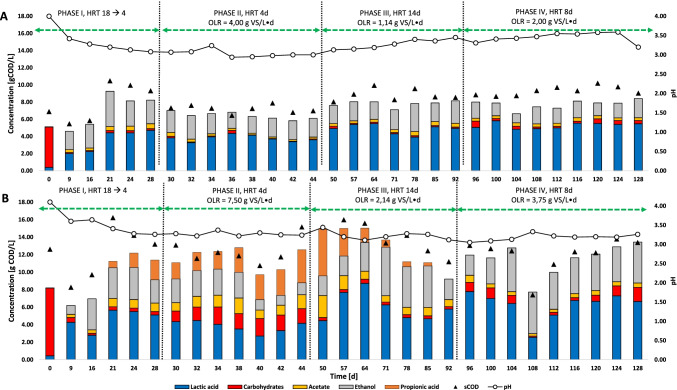
sCOD mass balance of R1 (**A**) and R2 (**B**) and pH profile of the effluent in every experimental phase at different HRT and OLR

pH profiles for R1 and R2 are shown in Fig. 3. The initial pH was 4.05 for both reactors. An immediate pH decrease was observed after the first batch cycle (day 9) reaching 3.42 in R1 and 3.60 in R2. The pH dropped slightly, but constantly, in both reactors during phase I with a subsequent stabilization until day 34 around 3.10 and 3.25, respectively. After a small increase on day 34 (probably due to fresh FW utilization), the pH dropped again in R1 settling at 2.95–3.00 until the end of phase II. On the contrary, R2 pH was stable in phase II fluctuating between 3.22 and 3.30. From phase III and for all phase IV, the pH value in R1 continuously increased until a final value of 3.59 was reached. For R2, the pH instead showed an oscillating behavior in phase III (3.44–3.12) with a subsequent stabilization at pH 3.20 during phase IV.

#### Fermentation profile

Figure 3 shows the composition of the effluent in R1 and R2 per phase expressed as g COD/L during the 128 days of operation. CO_2_ was the only biogas produced in every phase for both reactors, no H_2_ or CH_4_ was detected.

##### Phase I

The HRT in phase I was gradually reduced from 18 to 4 days in order to progressively increase the organic loads in ingress.

Reactor R1 showed a high consumption of carbohydrates with LA concentrations reaching a final value of 4.43 g COD/L. Apart from LA, ethanol was the other main fermentation product detected at a stable concentration of 4.11 g COD/L from day 21 onwards. VFAs production was almost inhibited in reactor R1 with some traces of acetate produced (0.57 g COD/L) (Fig. 3A).

Similar to reactor R1, carbohydrates were completely consumed in reactor R2 with resulting in a LA concentration of 5.12 g COD/L by the end of phase I. Ethanol was similarly produced starting at day 16 at a concentration of 3.55 g COD/L. Interestingly, from day 21 onwards, reactor R2 started producing propionic acid with simultaneous consumption of ethanol both reaching a concentration of 2.24 and 1.94 g COD/L, respectively, at the end of phase I (Fig. 3B). Similar to R1, reactor R2 only produced minimal acetate concentrations of < 1.00 g COD/L (Fig. 3B). During this phase, protein analysis in both reactors was measured resulting in very low concentrations (< 50 mg/L) to almost below the detection limit of the instrument.

##### Phase II

In phase II, the HRT was set to 4 days with feeding every 2 days. The OLR in this phase was 4.0 g VS/L day in R1 and 7.5 g VS/L day in R2 (Table 2).

The carbohydrate concentration in R1 was in line with the one of phase I. The LA concentration in R1 fluctuated between 3.27 and 4.36 g COD/L before stabilizing at 3.60 gCOD/L from day 36 until the end of phase II (Fig. 3A). Ethanol was still present at a constant concentration of 2.26 (± 0.27) g COD/L (Fig. 3A).

On the other hand, R2 showed a higher residual carbohydrate concentration (1.70 ± 0.25 g COD/L), corresponding to 13.80% of the sCOD, indicating that the OLR was too high to allow the biomass to complete their degradation at such a low HRT. R2 showed a drop in the LA production in phase II. The LA concentration fell from 5.12 g COD/L (day 28) to 2.71 g COD/L on day 40 (Fig. 3B) with a subsequent increase to 4.16 g COD/L on day 44. Ethanol was slowly degraded passing from a concentration of 2.72 g COD/L on day 30 to 1.13 g COD/L on day 42. Ethanol and LA consumption brought a coincident propionic acid production that, starting from a concentration of 1.85 g COD/L on day 30, reached 5.38 g COD/L at day 50 (Fig. 3B). Acetate was still detected in this phase with a concentration of 1.28 (± 0.28) g COD/L.

##### Phase III

Phase III was characterized by an increase of the HRT to 14 days (feeding every 7 days). The choice was made because the low HRT set in phase II resulted in a reduction of the efficiency in LA production in both systems and a lower carbohydrate conversion rate in R2. In phase III, the OLR was 1.14 g VS/L day in R1 and 2.14 g VS/L day (Table 2).

The carbohydrate concentration in R1 was in line with the other two phases. The LA concentration increased constantly until day 64 reaching a maximum value of 5.49 g COD/L (Fig. 3A) with a subsequent stabilization. The ethanol concentration was steady for the entire phase with an average concentration slightly higher than phase II (2.39 ± 0.39 g COD/L). Acetate was still present in minor concentrations (0.38 ± 0.07 g COD/L).

In reactor R2, the increasing of the HRT allowed a better substate utilization with only 2.50% of sCOD as residual sugars (Fig. 3B). Similar to R1 reactor, the LA concentration increased until day 64 reaching 8.72 g COD/L in R2 (Fig. 3). Then, the LA concentration decreased to 5.77 g COD/L on day 92 at the end of the phase. The ethanol concentration increased from 1.36 g COD/L on day 44 to 5.54 g COD/L on day 71, followed by a large drop that resulted in a final concentration of 2.34 g COD/L (Fig. 3B). Another benefit that the high HRT had on the system was the complete inhibition in the VFAs production in R2. Phase III was characterized by a constant and fast decrease of the propionic acid accumulated during phase II. The propionic acid concentration was 5.38 g COD/L on day 50 and was completely depleted within 3 cycles in phase III, dropping to 0.56 g COD/L by day 78 and 0 by day 92 (Fig. 3B). Acetate was still detected at a residual concentration of 1.19 (± 0.60) g COD/L.

##### Phase IV

Phase IV started on day 92 setting an HRT of 8 days with feeding every 4 days. This choice was made since it had been noticed that almost 95% of the carbohydrates were degraded after 4 days from the start of the cycles during phase III, so to optimize the production rates, it was decided to feed the reactors every 4 days resulting in an OLR of 2.00 g VS/L day for R1 and 3.76 g VS/L day in R2.

The residual carbohydrates concentration was 0.37 (± 0.17) g COD/L in R1 representing 3.96% of the sCOD (Fig. 2A). The LA concentration reached 5.83 g COD/L on day 100 with a stabilization to 5.28 (± 0.32) g COD/L (Fig. 3A). The ethanol concentration decreased in R1 to 1.73 (± 0.33) g COD/L (Fig. 3A). Acetate was the only VFA detected in R1 with a lower concentration of 0.35 (± 0.03) g COD/L.

R2 showed a residual carbohydrate concentration of 0.80 (± 0.46) g COD/L counting for 6.69% of the sCOD. In this phase, the LA concentration had a general improvement. R2 showed an average concentration of 6.80 (± 0.42) g COD/L (Fig. 3B). Opposite to R1, ethanol increased in R2 to an average of 4.15 (± 0.87) g COD/L (Fig. 3B). Acetate was the only VFA also in R2 (0.47 ± 0.12 g COD/L).

#### Lactic acid production yield and kinetic parameters

**Fig. 4:**
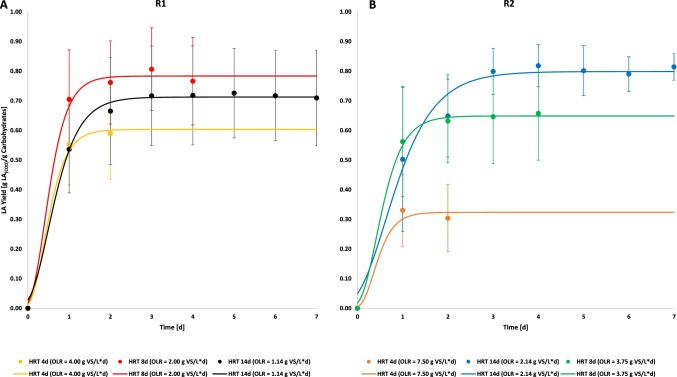
Cumulative LA production yields as a function of HRT and OLR in R1 (**A**) and R2 (**B**)

Figure 4 shows the cumulative LA yields expressed per unit of consumed carbohydrates of phases II, III, and IV calculated with Eq. 6. The Gompertz theoretical curves obtained by fitting the experimental data with Eq. 7 are also shown. The resulting calculated values of LA produced, carbohydrates consumed, LA yield and the Gompertz parameters (LA_Th_, k, and t_c_) are reported in Table 4. Phase I was excluded from the statistical yields analysis since the reactors were not operating in steady state conditions in that phase.

**Table 4:** Semicontinuous batch fermentation performance comparison between R1 and R2 at different HRT and OLR phases. Result parameters are shown in terms of carbohydrates degradation (CA_c_), lactic acid production (LA_p_), lactic acid production yield (LA_y_), along with the Gompertz equation parameter: kinetic constant (k), theoretical lactic acid yield (LA_th_), lag phase (t_c_), and r square (*R*^2^)

	R1			R2		
HRT* [day]	4	8	14	4	8	14
OLR* [g VS/L·day]	4.00	2.00	1.14	7.50	3.75	2.14
CA_c_ [g COD/L]	3.05 ± 0.53	3.37 ± 0.36	3.38 ± 0.23	4.78 ± 0.68	5.35 ± 1.04	5.90 ± 0.52
LA_p_ [g COD/L]	1.74 ± 0.32	2.54 ± 0.34	2.38 ± 0.48	1.45 ± 0.53	3.65 ± 0.52	4.57 ± 0.22
LA_y_ [g LA_(COD)_/g CA_(COD)_]	0.59 ± 0.15	0.80 ± 0.14	0.73 ± 0.17	0.33 ± 0.12	0.66 ± 0.16	0.82 ± 0.07
LA_th_ [g LA_(COD)_/g CA_(COD)_]	0.60 ± 0.02	0.78 ± 0.02	0.71 ± 0.01	0.32 ± 0.06	0.35 ± 0.01	0.80 ± 0.01
*K* [day^−1^]	3.40 ± 0.63	3.29 ± 0.41	2.41 ± 0.17	4.05 ± 1.49	3.43 ± 0.29	1.66 ± 0.22
*t*_c_ [day]	0.40 ± 0.03	0.40 ± 0.02	0.49 ± 0.02	0.36 ± 0.05	0.42 ± 0.02	0.62 ± 0.05
*R*^2^	0.993	0.994	0.997	0.977	0.996	0.988

^*^HRT, hydraulic retention time; *OLR*, organic loading rate

Both reactors show a wide range of production yields with the shifting of the HRT and OLR conditions. R1 (Fig. 4A), during phase II, showed a yield of 0.59 (± 0.15) g LA_(COD)_/g CA_(COD)_ that increased to 0.73 (± 0.17) g LA_(COD)_/g CA_(COD)_ during phase III and further to 0.80 (± 0.14) g LA_(COD)_/g CA_(COD)_ during phase IV. On the other hand, R2 (Fig. 4B) started in phase II with a yield of 0.33 g LA_(COD)_/g CA_(COD)_ reaching its highest value during phase III with 0.82 (± 0.07) g LA_(COD)_/g CA_(COD)_ and decreased again in phase IV stabilizing to a value of 0.66 (± 0.16) g LA_(COD)_/g CA_(COD)_.

As reported in Table 3, both reactors showed the highest k value at 3.40 day^−1^ (R1) and 4.05 day^−1^ (R2) during phase II, operating at a HRT of 4 days and OLR of 4.00 g VS/L·day and 7.50 g VS/L·day, respectively. The increase of the HRT in phase III (4 to 14 day) brought a slower kinetic with k values of 2.41 day^−1^ for R1 and 1.66 day^−1^ for R2. This was followed by a further increased in k value in phase IV (HRT 8 day) with 3.29 day^−1^ for R1 and 3.43 day^−1^ for R2.

### Microbial community development

**Fig. 5:**
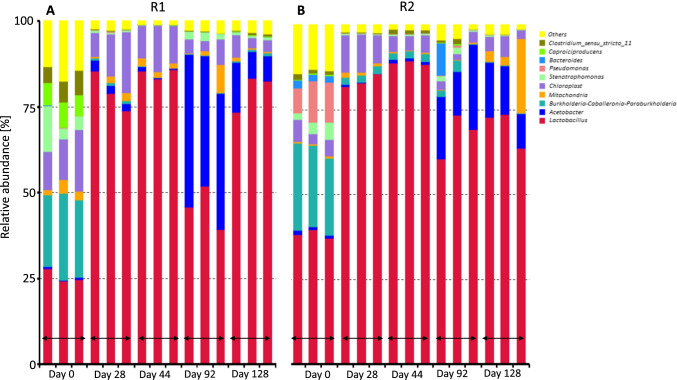
Relative abundance at genus level evolution during every phase of the experimental run in R1 (**A**) and R2 (**B**)

Figure 5 shows the evolution of the microbial profile for R1 (Fig. 5A) and R2 (Fig. 5B) at the end of every fermentation phase as % relative abundance (RA) at genus level. The results are shown in triplicates for every sampling day.

At the startup of the reactors (day 0), the microbial community was the results of the bacteria present in the inoculum and the endogenous FW microorganisms. As shown in Fig. 5, slight differences are present in the microbial RA between R1 and R2, likely due to the fact that R2 had a higher initial FW load. The initial composition for reactor R1 showed 25.66 (± 1.64) % *Lactobacillus*, 23.03 ± (1.73) % *Burkholderia-Caballeronia-Paraburkholderia*, 13.63 ± (3.02) % *Chloroplast*, 6.90 (± 4.61) % *Stenotrophomonas*, 6.43 (± 0.61) % *Caproiciproducens*, and 6.00 (± 1.02) % *Clostridium.* The initial R2 microbial community was composed of 38.39 (± 1.01) % *Lactobacillus*, 24.27 (± 1.23) % *Burkholderia-Caballeronia-Paraburkholderia*, 10.47 ± (2.10) % *Pseudomonas*, and 4.73 (± 1.47) % *Chloroplast*.

By the end of phase I (day 28), *Lactobacillus* was the main strain dominating the microbial community in both R1 and R2 with an increased RA of 79.41 (± 4.75) % and 83.54 (± 1.57) %, respectively. This trend was similar at the end of phase II (day 44) in which their relative abundance reached the maximum with values of 84.89 (± 1.19) % for R1 and 88.9 (± 0.44) % for R2. In the same period, the other main strains shown on day 0 almost disappeared with just *Chloroplast* present at < 5% RA. At the end of phase III (day 92), a drastic reduction in the RA of *Lactobacillus* was observed for both reactors decreasing to 45.64 (± 5.51) % for R1 and 67.78 (± 5.32) % for R2. Coincidentally, with the decrease in *Lactobacillus*, the growth of the *Acetobacter* strain was detected at 40.83 (± 2.83) % RA for R1 and 18.77 (± 4.96) % RA for R2. The last phase (day 128) highlights a difference in the microbial community progression between the two reactors: R1 *Lactobacillus* RA bounced back increasing to a final value of 79.79 (± 4.51) %; while in reactor R2 only showed a slight increase from phase III resulting to a final RA of 70.15 (± 4.47) %. *Acetobacter* was still present at the end of the run at a RA o f9.97 (± 3.28) % for R1 and 13.50 (± 2.54) % for R2.

## Discussion

### LA fermentation in very acidic environment

This study shows that semicontinuous LA fermentation of FW is feasible under uncontrolled and acidic pH condition. This was evident in the semicontinuous run where both reactors had a pH below the LA pKA value of 3.78 and yet were able to consistently produce LA as the main metabolite through the entire experimental run. The highest registered LA concentration was reached in R2 during phase III at 8.72 g COD/L and a pH of 3.11 (Fig. 3B). In addition to achieving the highest LA concentration, this phase also recorded the highest LA production yield at 0.82 g LA_(COD)_/g CA_(COD)_ (Table 4). Furthermore, R1 maintained a high stability in terms of LA concentration (3.27–5.83 g COD/L) and production along the experimental run with the pH varying from 2.95 (phase II) to 3.59 (phase IV) showing that LA fermentation occurs even at a pH lower than 3.00. These results are in line with our previous batch screening work [26] where a pH < 4.0 was found to be the most suitable for LA production and accumulation.

The optimal pH for LA production is still a debated question with several studies reporting opposing views. However, it can be noted that the best pH range largely depends on the type of raw substrate, inoculum, and reactor configuration used [30]. Currently, there are very few papers published on LA production at low pH in a semicontinuous/continuous mode using FW as the substrate. Feng et al. [31] and Itoh et al. [24] reported similar trends to this study in LA production with response to the pH condition. The study of Feng et al. [31] reports how the final metabolite composition of the fermentation broth changes depending on the pH in batch reactors. The fermentation of FW using anaerobic sludge as inoculum under mesophilic conditions was reported by these authors. They state that stable homolactic fermentation occurs when the pH was between 3.2 and 4.5. They found that at pH 3.2, LA was the main product accounting for 32.4% of the sCOD with a concentration of 5.7 g COD/L. However, further pushing the pH to 4.0, they reached additional production of LA, reaching 13.4 g COD/L representing 53.9% of the sCOD [30]. Finally, they showed that for a pH above 4.5, the LA concentration decreased to 0.6 and 0.3 g COD/L at pH 5.00 and 6.00, respectively, with a subsequent improvement in the VFAs generation. Similarly, Itoh et al. [24], using glucose as the main substrate, reported an optimal pH of 3.50 for LA fermentation with a LA production of 4.1 g COD/L after 18 h from the start of the experiment in mesophilic conditions [24].

One of the main advantages of working at low pH is that *Lactobacillus* strains are among the few microorganisms able to survive and resist the acidic stress caused by the acidogenesis [32]. This results in an inhibitory effect on other organic acid producers [24, 32] that compete with LAB for the carbohydrates or that could use the LA itself as primary substrate for bioconversion to VFAs such as propionic [33] or butyric acid [34]. This was shown in the study of Xu et al. [35] where a semicontinuous configuration was used to perform LA fermentation from FW and activated waste sludge co-digestion. The reactor pH was set and controlled at 9.0, according to the findings of Zhang et al. [36], and HRT 4 days. The LA concentration increased rapidly in the first 5 days reaching 31.7 g/L and then dropped to below the detection limit by day 12 with a subsequent production of VFAs [37]. Methanogenic activity is also stopped since a more neutral pH is required for methane production.

On the other hand, there are also some risks associated to working at these acidic conditions [32]. One of them is that high concentrations of LA in its undissociated form can be inhibitory on LAB metabolism since LA can enter the cells and an over-accumulation could bring membrane destabilization or destruction [18, 32]. Moreover, enzymatic activity can be restricted and microbial growth rates can be slowed down [38]. This can be solved by providing a continuous LA extraction process to avoid its accumulation in the fermentation tank.

From an applicative point of view, a low pH means that no alkalinizing agents are required to maintain neutrality during the fermentation process, this has a direct effect on the fermentation costs since the reagents count 15% of the total expenses [23]. A problematic aspect of using pH corrector reagents is the LA conversion to solid lactate salt (calcium lactate or sodium lactate) that must be acidified and then purified back to LA in the downstream phase with a net production of gypsum as waste [32]. Low pH operation leads to a simplification of the downstream processes since LA can be extracted directly from the fermentation broth in liquid form. Nowadays, the downstream process for LA extraction and purification accounts for 40–70% of the total cost and represents the main cost along with the substrate [39].

### Effect of OLR and HRT on LA production

**Fig. 6:**
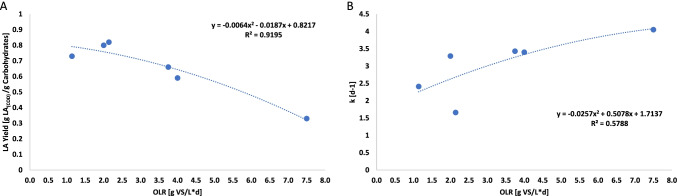
Variation of the maximum yield (**A**) and the kinetic constant (**B**) from both reactors in function of the OLR

Figure 6A shows the relation between production yield with the OLR. The graph was obtained by merging the six different OLRs of the two reactors and their resulting LA yield/production. It can be highlighted that both reactors performed better at low OLR and a subsequent high HRT. As can be noticed from the LA production yield, the trend has a parabolic pattern and has an optimum OLR around 2.00 g VS/L day followed by a drop when the OLR increases

The two reactors operated at similar OLR when different HRT were set (due to the diverse initial solid concentration): R1 had an OLR of 2.00 g VS/L day at HRT 8 days while R2 was operating at 2.14 g VS/L day CA and HRT 14 days. The difference in the HRT did not affect the performance since the production yields were 0.80 (± 0.14) g LA/g CA and 0.82 (± 0.07) g LA/g CA in R1 and R2, respectively. The independency of the yield from the HRT is confirmed also when R2 and R1 operated at an OLR of 3.75 g VS/L day and 4.00 g VS/L day, respectively, and their correspondent yields were 0.66 (± 0.16) and 0.59 (± 0.15) g LA/g CA where the HRT was 4 days for R1 and 8 days for R2. Regarding the kinetic constant (k), it follows an opposite trend compared to the yield: the higher the OLR (and the HRT low), the higher k is, indicating a faster velocity of carbohydrate conversion to LA. The highest value of k of 4.05 (± 1.49) day^−1^ was achieved in reactor R2 at an OLR 7.50 days as shown in Fig. 6B.

Several studies report the influence of the OLR and HRT for LA production. Table 5 reports the conditions (pH, OLR, and HRT) of the best scenarios in terms of LA concentration and LA yields obtained using semicontinuous systems. Tang et al. [22] tested three different OLRs for the LA conversion from FW. The OLRs adopted were 14, 18, and 22 g TS/L•d with a fixed HRT of 5 days. Their results showed a similar trend with the one shown in this study (Fig. 6A), yields decreased from 0.44 g LA/g TS (OLR 14 g TS/L day) to 0.31 g LA/g TS (OLR 22 g LA/g TS) [22]. Luongo et al*.* [12] studied the influence of the OLR and the HRT in a repeated batch reactor configuration for the LA fermentation of cheese whey. Three OLRs were investigated by feeding the same concentration of cheese whey at different HRTs. The latter was constantly reduced from 4 days to 1 day with a subsequent increasing of the OLR from 19.6 to 49.0 g VS/L day. As can be noticed, the OLR adopted by Luongo et al. was higher than the one tested in this study; however, this is mainly because the cheese whey solid fraction is lower compared to FW. They reported higher conversion yields when the OLR was low passing from 0.33 g LA_(COD)_/g COD at an OLR of 19.6 g VS/L day compared to 0.23 g LA_(COD)_/g COD at an OLR 49.0 g VS/L day [12]. A repeated batch reacotr system was also adopted by Xu et al. [34] to investigate the semicontinuous LA production from FW at pH 9.0 and HRT 4 days. In the comparative study of the batch cycles composing the experimental run, they reported an increasing LA concentration in the first three days of every cycle followed by a LA consumption with net production of VFAs (mainly acetate and propionate). The maximum LA concentration was 28.1 g COD/L with a yield of 0.72 g LA/g COD.

**Table 5:** Comparative table of the highest of lactic acid yields and maximum lactic acid concentrations reported in the literature using either semicontinuous or continuous systems at different fermentation conditions (pH, HRT, and OLR)

Substrate	Reactor configuration	pH condition	HRT ** [day]	OLR**	Max LA conc [g COD/L]	LA* yield	Reference
FW*	RBR	6.0	5.0	14 [g VS/L·day]	29.0	0.76 [g/g COD]	[22]
CW*	RBR	Uncontrolled	2.0	32.4 [g VS/L·day]	20.1	0.37 [g/g COD]	[12]
FW + WAS*	RBR	9.0	4.0	10 [g COD/L·day]	28.7	0.72 [g/g COD]	[34]
Glucose	CSTR	3.5	1.0	10 [g C_6_H_12_O_6_/L·day]	4.3	0.44 [g/g CA]	[24]
CW	CSTR	5.5	0.5	20 [g COD/L·day]	5.5	0.62 [g/g COD]	[36]
FW	RBR	Uncontrolled	14.0	2.14 [g VS/L·day]	8.7	0.82 [g/g CA]	This study

^*^FW, food waste; *CW*, cheese whey; *WAS*, waste activated sludge; *LA*, lactic acid

^**^HRT, hydraulic retention time; *OLR*, organic loading rate

As can be seen from these examples, reactor performances tend to decrease when adopting a high OLR and low HRT, which is similar to the findings of this study. Within these conditions, the total solid concentration of the reactor increases the viscosity of the fermentation broth which leads to a reduction in mass transfer between the substrate and the microorganisms [40]. The studies reported above showed not only less LA production but also a limited carbohydrate degradation with high unconsumed residual substrate in the effluent indicating a low hydrolysis rate [36, 37, 40]. Working at high OLR would be auspicious from an industrial point of view since high load means lower working volume and, therefore, a cheaper process. However, the decrease in the conversion efficiency and production yield as a consequence of the high OLR obtained in this study and highlighted in the literature suggests that a compromise among these factors is needed.

Another reason that can bring a drop in the conversion of sugars for LA production is substrate inhibition [18]. Substrate inhibition occurs when substrate concentrations raise above a critical limit creating a stress environment for bacteria that cause long acclimation periods, osmotic stress, and cell lysis. This has as main consequence the reduction of sugar utilization and the decrease of the conversion yields [18, 41]. Avoiding substrate inhibition is still a main challenge of LA research. One of the proposed methods to solve it is to isolate specific bacterial strains [18, 42]. In the study of Zhang et al. [42] different *Lactobacillus* strains were isolated and tested at different glucose concentrations (6 – 115 g/L) to investigate which was the most resistant one. Their results show a drop in the substrate utilization occurred at the highest glucose concentrations (85 and 115 g/L). Despite that the LA conversion yield (expressed in g LA/g CA) was almost stable for every isolated strain, with the maximum achieved with *Lactobacillus reuteri DTUAT-04* of 0.70 g LA/g CA. Despite using pure isolated cultures and a simple substrate as glucose, the study of Zhang et al. achieved lower production yields than the one reported in this study [42].

The contribution of the proteins content in the FW fermentation for LA production was considered negligible as the protein concentration at the beginning of every cycle was very low (< 50 mg/L). The metabolic role of proteins during LA fermentation has yet to be studied and, to the best of the authors knowledge, no publication reports LA production impacts from protein. In the study of Alibardi and Cossu (2016) [43], the authors state that the proteins content can influence the fermentation for their high nutrient concentration that can favor the bacterial development and that 90% of protein degradation during fermentation occurs through the Stickland reaction pathway in which VFAs (especially butyrate and acetate) are the main bioproducts.

### Microbial community evolution and fermentation pathways

To the best of the authors’ knowledge, this study is the first to investigate the use of yogurt as an inoculum in LA fermentation. Anaerobic microorganisms have a key role in the final metabolite composition of the fermentation effluents. *Lactobacillus* are reported to be one of the main species responsible for direct LA production [40]. They work following three main pathways in which LA is the main metabolite of their carbohydrate digestion:

8 $${\mathrm{C}}_{6}{\mathrm{H}}_{12}{\mathrm{O}}_{6}\to 2{\mathrm{CH}}_{3}\mathrm{CH}\left(\mathrm{OH}\right)\mathrm{COOH }$$

9 $${\mathrm{C}}_{6}{\mathrm{H}}_{12}{\mathrm{O}}_{6} \to {\mathrm{CH}}_{3}\mathrm{CH}\left(\mathrm{OH}\right)\mathrm{COOH }+ 1.5{\mathrm{CH}}_{3}\mathrm{COOH}$$

10 $${\mathrm{C}}_{6}{\mathrm{H}}_{12}{\mathrm{O}}_{6} \to {\mathrm{CH}}_{3}\mathrm{CH}\left(\mathrm{OH}\right)\mathrm{COOH }+\mathrm{ C}{\mathrm{O}}_{2} +\mathrm{ C}{\mathrm{H}}_{3}{\mathrm{CH}}_{2}\mathrm{OH}$$

Equation 8 represents the homolactic fermentation (Embden-Meyerhof-Parnas (EMP) pathway) in which all the glucose mass is theoretically converted to LA [44], while Eq. 9 and Eq. 10 represent the heterolactic fermentation in which for every mol of glucose degraded a mol of lactate is produced along with acetate (Bifidus pathway) [44, 45] or ethanol (Phosphoketolase pathway) [44]. The results of the initial screening of batch tests for the choice of the inoculum suggests that in the B2 incubation, using yogurt, heterolactic fermentation (Eq. 10) occurred since LA and ethanol were produced almost in equal amounts with a ratio 1:1 in terms of g LA_(COD)_/g Ethanol_(COD)_ (Fig. 2–B2). The presence of ethanol was detected also in the semicontinuous run in both reactors despite LAB are known for their strictly homofermentative metabolism [18]. This can mainly be due to two factors: the first is that FW could have, among its endogenous microbial ecology, some ethanol producers that affect the *Lactobacillus* present in the yogurt. The second aspect is that LAB could shift their metabolism based on the number of carbons of the sugars there are utilizing. The review of *Adbel Rahman and Sonomoto* [18] reports that several lactobacillus strains can change their metabolism to heterofermentative when pentose sugars are used as the substrate. FW has a high heterogeneity in its carbohydrate composition and the formation of ethanol can be due to the degradation of the pentose sugars present in the FW itself.

The microbial community composition matches with the organic acid spectrum found in the fermentation broth (Fig. 3) in which LA is the main product highlighting that *Lactobacillus* strains were the dominant microorganisms inside R1 and R2 for most of the experimental run. The maximum RA was reached at the end of phase II (day 44) in which *Lactobacillus* accounted for 84.9 and 88.9% of the total microbial community. This maximum was observed in both reactors when the HRT was 4 days. With the change of HRT from 4 to 14 days during phase III (day 92), a large drop in the *Lactobacillus* RA can be noticed in R1 and R2, decreasing to 45.4% and 67.8%, respectively, with a contemporaneous growth of the *Acetobacter* RA (Fig. 5). The *Lactobacillus* RA raised again in the last phase IV in which the HRT was set to 8 days. The similar trend in the *Lactobacillus* evolution for both reactors indicates that the microbial structures present in the reactor depend also on the HRT. Focusing on the *Lactobacillus* strain, at the lower HRT, they were the only microorganisms able to reproduce quickly enough to keep their concentration high, highlighting their faster growth kinetics compared to the VFAs producers. The latter were likely washed out from the reactor when the HRT was set at 4 d, but their concentration dramatically increased when the HRT was set to its maximum value of 14 days (Fig. 5). Interestingly, the hydrolytic microbial population, such as *Actinobacteria*, was abstent in the microbial population, potentially due to the low pH which weakened the activity of hydrolysis [46, 47].

*Acetobacter* was the strain which grew more when the HRT was changed from 4 to 14 days in phase III (Fig. 5), reaching a RA of 40.8% and 18.7% in R1 and R2, respectively. *Acetobacter* strains are responsible for the formation of acetic acid from different substrates. This strain is reported in different studies as able to form acetic acid from starting substrates such as carbohydrates, ethanol, and hydrogen plus carbon dioxide [48, 49]. Despite the high abundance of *Acetobacter* in the fermentation broth, the concentration of acetic acid in phase III was relatively low reaching only 0.48 g COD/L and 2.51 g COD/L in R1 and R2, respectively. This is likely due to the low operational pH of the reactors that was inhibiting the *Acetobacter* strains, responsible for acetogenesis, from converting longer chain products to acetate.

A peculiar trend was observed for R2 where the production of propionic acid was observed from mid-phase I to mid-phase III. Propionic acid accumulated in the system from days 21 to day 44 reaching the maximum on day 50 at 5.38 g COD/L (Fig. 3B). This was followed by a constant and quick decrease in its concentration during phase III when the HRT was 14 days. It is important to highlight that during propionic acid accumulation, there was a simultaneous reduction in the LA concentration. Formation of propionic acid is common during fermentation and several strains are reported as propionate producers. Three different biological pathways are involved in propionic acid production: the succinate pathway, acrylate pathway, and propanediol pathway [50]. The first two are likely the pathways that performed the high propionic generation inside R2. In the first pathway propionate is formed from carbohydrates (glucose, lactose, and glycerol), while in the second lactate is the main substrate that is converted directly in propionic acid [50]. Some *Firmicutes* and *Bacteroides* microorganisms can perform these two pathways: *Veillonella* [51] and *Prevotella* [52] are reported to be able to produce propionate from lactate and glucose with a production yield of 0.59 and 0.30 g /g COD, respectively. These two strains were both found in the inoculum and all fermentation runs with a RA < 1% (Fig. 5).

## Conclusions

Semicontinuous fermentation of FW at acidic conditions (pH < 4) was feasible for LA production as the main metabolite using yogurt as inoculum. The results show that LA was still formed under the acidic conditions set in the reactors with pH 2.95 as the lowest pH registered at which LA was still produced. In terms of fermentation efficiency, LA reached the highest concentration of 8.72 g COD/L and yield of 0.82 g LA/g CA at an operational parameter of 14-day HRT and 2.14 g VS/L day OLR. The LA production yield decreased when increasing the OLR and reducing the HRT. *Lactobacillus* dominated the microbial community for the largest part of the experimental run, especially at the minimum HRT (4 days), indicating that their kinetic growth is faster than that of VFAs producers and that low pH does not inhibit their fermentation activity. The obtained results provide a base for a possible industrial application merging the possibility to decrease the food waste impact on the environment and its valorization with the necessity of the cost reduction that LA production requires nowadays to make PLA competitive with fossil-based plastics. Despite the potentiality of the process, some key aspects still have to be addressed by future research like the possibility to increase the initial substrate concentration entering the reactor solving the problems of substrate inhibition and the possibility of a continuous lactic acid extraction to avoid its overaccumulation in the system that could induce bacterial inhibition.

## Data Availability

The data will be provided on request to the corresponding author.
